# A Simple Polymicrobial Biofilm Keratinocyte Colonization Model for Exploring Interactions Between Commensals, Pathogens and Antimicrobials

**DOI:** 10.3389/fmicb.2020.00291

**Published:** 2020-02-26

**Authors:** Elena Jordana-Lluch, Vanina Garcia, Alexander D. H. Kingdon, Nishant Singh, Cameron Alexander, Paul Williams, Kim R. Hardie

**Affiliations:** ^1^Centre for Biomolecular Sciences, School of Life Sciences, University of Nottingham, Nottingham, United Kingdom; ^2^School of Pharmacy, University of Nottingham, Nottingham, United Kingdom

**Keywords:** polymicrobial biofilm, skin infections, *Pseudomonas aeruginosa*, *Staphylococcus*, *Micrococcus luteus*, quorum sensing, keratinocyte, antimicrobial

## Abstract

Skin offers protection against external insults, with the skin microbiota playing a crucial defensive role against pathogens that gain access when the skin barrier is breached. Linkages between skin microbes, biofilms and disease have not been well established although single-species biofilm formation by skin microbiota *in vitro* has been extensively studied. Consequently, the purpose of this work was to optimize and validate a simple polymicrobial biofilm keratinocyte model for investigating commensal, pathogen and keratinocyte interactions and for evaluating therapeutic agents or health promoting interventions. The model incorporates the commensals (*Staphylococcus epidermidis* and *Micrococcus luteus*) and pathogens (*Staphylococcus aureus* and *Pseudomonas aeruginosa*) which form robust polymicrobial biofilms on immortalized keratinocytes (HaCat cells). We observed that the commensals reduce the damage caused to the keratinocyte monolayer by either pathogen. When the commensals were combined with *P. aeruginosa* and *S. aureus*, much thinner biofilms were observed than those formed by the pathogens alone. When *P. aeruginosa* was inoculated with *S. epidermidis* in the presence or absence of *M. luteus*, the commensals formed a layer between the keratinocytes and pathogen. Although *S. aureus* completely inhibited the growth of *M. luteus* in dual-species biofilms, inclusion of *S. epidermidis* in triple or quadruple species biofilms, enabled *M. luteus* to retain viability. Using this polymicrobial biofilm keratinocyte model, we demonstrate that a quorum sensing (QS) deficient *S. aureus agr* mutant, in contrast to the parent, failed to damage the keratinocyte monolayer unless supplied with the exogenous cognate autoinducing peptide. In addition, we show that treatment of the polymicrobial keratinocyte model with nanoparticles containing an inhibitor of the PQS QS system reduced biofilm thickness and *P. aeruginosa* localization in mono- and polymicrobial biofilms.

## Introduction

Skin is the largest organ of the body and functions as a physical barrier against external insults, such as toxins or pathogenic microorganisms (Parlet et al., 2019). Skin is principally composed of an epidermis and underlying dermis with keratinocytes constituting 90–95% of the upper epidermal layer nearest to the colonizing microbiota. Depending on their differentiation state, keratinocytes are arranged in stratified layers and are potent sources of antimicrobial peptides and cytokine/chemokine signals. Keratinocytes orchestrate the formation of the stratum corneum which physically separates the viable layers of the cutaneous epithelium from surface microbes. The stratum corneum is a waxy waterproof composite containing flattened corneocytes and interlocking keratinocyte-derived lipids and granules that forms a tight mechanical barrier. Despite this seemingly inhospitable niche that is acidic, lipid dense and lacking in nutrients, direct contact with the environment results in skin becoming colonized by a diverse community of bacteria, fungi, viruses and mites, for which the skin offers a variety of stable niches with different environmental conditions and nutrients (Grice and Segre, 2011; Brandwein et al., 2016; Byrd et al., 2018; Erin Chen et al., 2018). Skin also acts as an immunological barrier that distinguishes between commensals and harmful microbes (Negrini et al., 2014). The commensal microbiota play an important role by “educating” the innate immune system to mount a response against antigens produced by pathogens, but not by commensals (Leech et al., 2019). Furthermore, commensals secrete proteins and other metabolites that modulate the virulence of pathogens or give the commensals a selective advantage to outcompete the pathogens (Cogen et al., 2008; Grice and Segre, 2011; Chen and Tsao, 2013; Bosko, 2019; Parlet et al., 2019). For instance, *Staphylococcus epidermidis* autoinducing peptide (AIP) signal molecules inhibit the *Staphylococcus aureus agr* system and hence exotoxin production (Otto et al., 2001) whereas *Microccus luteus* enhances *S. aureus* pathogenesis (Boldock et al., 2018).

Skin damage facilitates the entry of pathogenic bacteria resulting in an infected wound. Chronic wound infections, cost the United Kingdom healthcare service over £1 billion per year (Percival et al., 2012). Due to their links with predisposing conditions including diabetes and obesity, such chronic wound infections are increasing in prevalence. Infected surgical wounds increase the average hospital stay by 10.2 days compared with those that heal without complications. Extended periods of bacterial colonization in patients receiving antibiotics also create selection pressures that may allow resistant pathogens to emerge, impairing treatment and further delaying healing (Bowler et al., 2001).

The chronicity of wound infections is linked to biofilm formation because the extracellular matrix protects bacteria against host defenses and antimicrobial agents (Snyder et al., 2017). Biofilm related persistent infections account for 65–80% of all infections (Macià et al., 2014). Furthermore, such biofilms tend to be polymicrobial, which worsens prognosis (Peters et al., 2012). *Staphylococcus aureus* and *Pseudomonas aeruginosa* commonly infect chronic wounds and are often isolated from the same infection site (Deleon et al., 2014; Serra et al., 2015). Both species have developed intricate regulatory networks to achieve evasion, counter-inhibition and suppression of the other bacterial species to enable them to co-exist in the same niche (Hotterbeekx et al., 2017). Furthermore, the growth of the two pathogens together can offer mutual benefit through increased biofilm production, gentamicin tolerance and more severe infection (Deleon et al., 2014).

The regulation of biofilm development is complex, involving diverse transcriptional and post-transcriptional mechanisms. These include the population density dependent cell-cell communication network known as quorum sensing (QS) (Atkinson and Williams, 2009). The QS network of *P. aeruginosa* integrates three systems, *las*, *rhl*, and *pqs* (Lee et al., 2013), whereas *S. aureus* employs the accessory gene regulator (*agr)* QS system (Gordon et al., 2013; Bronesky et al., 2016). QS regulates the expression of diverse virulence genes (Njoroge and Sperandio, 2009), and thus QS inhibition has been widely investigated as an alternative to antibiotics to tackle specific pathogens given that interference with signaling would allow attenuation of virulence without compromising bacterial viability, thereby reducing the likely selection of resistance (Njoroge and Sperandio, 2009; Romero et al., 2012; Soukarieh et al., 2018; Shaaban et al., 2019). A notable added benefit is that QS inhibition may reduce biofilm maturation such that susceptibility to antibiotics and host defenses is enhanced. Recently, the usefulness of encapsulating a quorum sensing inhibitor (QSI) targeting the PqsR receptor of *P. aeruginosa* has been demonstrated (Singh et al., 2019). In an alginate nanoparticle delivery system, the QSI reduced *P. aeruginosa* biofilm development on keratinocytes, and when delivered in combination with an antibiotic and fully cleared *P. aeruginosa* biofilms in an *ex vivo* pig skin model (Singh et al., 2019).

Several *in vitro* models describing polymicrobial biofilms, including those pathogens most relevant to skin infections, have been developed (Coenye and Nelis, 2010; Negrini et al., 2014). The main drawback of these models is the lack of a host component (Ganesh et al., 2015; Roberts et al., 2015). *In vivo* (mice or porcine) models allow long term infection and mimic the chronicity of wounds (Ganesh et al., 2015). However, these models suffer from ethical limitations, especially when high-throughput analysis to test several anti-biofilm compounds needs to be performed. Thus, a simple, robust 2D polymicrobial model of skin infection that would facilitate the investigation of the interactions between skin colonizing bacteria (both commensal and pathogen) and to validate interventions, such as the impact of a QSI (Singh et al., 2019) is highly desirable. Here, we describe and validate a proof of concept model incorporating both commensals (*S. epidermidis* and *Micrococcus luteus*) and pathogens (*S. aureus* and *P. aeruginosa*) using HaCat cells (immortalized keratinocytes) as the model “skin” substrate. This simple skin model is shown to be useful for investigating commensal, pathogen and keratinocyte interactions and for evaluating therapeutic agents or health promoting interventions. We exemplify this by evaluating the impact of mutating the *S. aureus agr* QS system or delivering an anti-pseudomonal QSI nanoparticle on the polymicrobial biofilm and keratinocyte community.

## Materials and Methods

### Bacterial Strains and Growth Conditions

The bacterial strains, plasmids and antibiotics used are listed in Table 1. All bacteria were grown at 37°C in LB (*P. aeruginosa*) or BHI (*S. aureus*, *S. epidermidis*, and *M. luteus*), and shaken at 200 rpm when required. Plasmids including pSB2019 (carrying GFP protein) (Qazi et al., 2001) was transformed into the group I *agr S. aureus* strain SH1000 (Doherty et al., 2006) and *S. epidermidis* 1457 (Galac et al., 2017) as described elsewhere (Monk et al., 2012). *S. aureus* SH1000 Δ*agr* was constructed by transducing the Δ*agr*:*tetM* cassette from strain RN6911 (Novick et al., 1993) using phage phi ϕ11 as described previously (McVicker et al., 2018).

**TABLE 1:** Strains used in this study.

Microorganism	Strain	Plasmid	Antibiotic resistance	References
*Micrococcus luteus*	2665		Furazolidone, Nalidixic acid, Colistin (all at 10 μg/ml)	Rokem et al., 2011
*Staphylococcus epidermidis*	1457		Nalidixic acid, Colistin (all at 10 μg/ml)	Galac et al., 2017
*Staphylococcus epidermidis*	1457	pSB2019-gfp	Chloramphenicol, Nalidixic acid, Colistin (all at 10 μg/ml)	This study
*Staphylococcus aureus*	SH1000	pSB2019-gfp	Chloramphenicol, Nalidixic acid, Colistin (all at 10 μg/ml)	This study
*Staphylococcus aureus*	SH1000	pmKAT	Erythromycin (20 μg/ml) Nalidixic acid, Colistin (both at 10 μg/ml)	This study
*Staphylococcus aureus*Δ*agr*	SH1000		Tetracycline, Nalidixic acid, Colistin (all at 10 μg/ml)	This study
*Pseudomonas aeruginosa*	PAO1-Nottingham	pME6032-mCherry	Tetracycline (125 μg/ml)	Heeb et al., 2000;
				Ortori et al., 2011

### 2D Infection Model

#### HaCat Cells

Immortalized keratinocytes (HaCat, Culture Cell Lines, CLS Gmbh) were used as the “skin” substrate for the infection model. Cells were expanded in T75 flasks (Corning), in RPMI-1640 with phenol red supplemented with 10% v/v heat-inactivated foetal bovine serum (FBS), 1% v/v L-glutamine (200 mM) and 1% v/v penicillin (10,000 units/mL)/streptomycin (10 mg/mL) until 80% confluent. After removing the growth medium, cells were trypsinised using 7 ml of a solution containing 0.5 g/L trypsin/0.02 g/L EDTA. Trypsinization was stopped by adding 7 ml of heat-inactivated FBS. Cells were pelleted (5 min, 300 × *g*) and resuspended in 2 mL of RPMI supplemented with phenol red and seeded at 45,000 cells/cm^2^ in an eight well micro-slide Ibitreat chamber (ibidi, GmbH, Martinsried, Germany). When 100% confluent (approximately 90,000 cells/cm^2^), cells were washed three times by adding/removing 300 μL of Dulbecco’s Phosphate Buffered Saline (DPBS), prior to infection. HaCat cells were stained using either CellTracker (deep red) before infection or CellMask (deep red) prior to confocal imaging (both from Thermo Fisher Scientific), following the manufacturer’s instructions.

#### Bacteria

Separate overnight cultures of *S. aureus*, *S. epidermidis*, and *M. luteus* (day 1 of infection) were diluted 1:10 with fresh BHI (for both staphylococcal species) or 1:5 (for *M. luteus*) and further incubated at 37°C, 200 rpm until an OD_600_ 1–1.5. For *P. aeruginosa* (day 2 of infection), the overnight culture was diluted 1:5 with fresh LB and further incubated (37°C, 200 rpm) until an OD_600_ 0.8–1 was reached. For all bacteria, 1 ml of culture was pelleted for 1 min at 13,000 rpm and washed with Phosphate Buffered Saline (PBS, pH7.4). The cultures were subsequently resuspended in RPMI-1640 without phenol red to an OD_600_ 0.01 in a final volume of 5 mL and further diluted 1:1,000. *S. aureus* and *P. aeruginosa* were further diluted to reach a 1:10,000 and 1:100,000 dilution, respectively. For the polymicrobial studies, the volume required to obtain an OD_600_ 0.01 for each bacterial species was added together, adjusting the volume of RPMI accordingly and further diluted as above. A total of 150 μL were added to each well containing confluent HaCat cells (day 1 of infection) or HaCat cells and the Gram-positive bacteria (day 2 of infection), setting up 2 repeats per condition. Infected cells were incubated at 37°C, with 5% v/v CO_2_ and 95% humidity. Planktonic cells and growth medium were carefully removed and 100 μl of PBS were added to each well to avoid desiccation during confocal imaging (CLSM, LSM 700 Carl Zeiss, Germany).

#### Image Analysis

An average of 4–5 Z-stack images per well (8–10 per condition) were taken. Biomass, average thickness and surface area of the biofilms were quantified using COMSTAT2 software (Heydorn et al., 2000), applying automatic thresholding (Otsu’s method) and without connected volume filtering. Three biofilm parameters were analyzed. bio-volume (which represents the overall volume of the biofilm and provides an estimate of its biomass), average thickness (which provides a measure of the spatial size of the biofilm) and surface area (which calculated the area of the biomass surface exposed to the environment). A total of four independent experiments, with eight images per condition were analyzed.

#### Statistical Analysis

GraphPad Prism 7 software was used for graphical representation and statistical analysis. Quantitative variables were compared using a ratio paired two-tailed Student’s t. *p-*values < 0.05 were considered statistically significant.

### Biofilm Bacterial Viable Counts

Biofilms were prepared in 96 well plates by adding 200 μl of the diluted cultures prepared as outlined above and incubated for a total of 40 h (but where included, *P. aeruginosa* was added after 20 h incubation). Biofilms were disrupted by sonication for 5 min and thoroughly resuspended by pipetting. Thirty microlitres of resuspended biofilm were added to 270 μl PBS in a 96 well plate and diluted up to 10^–8^. Five microliters were plated onto agar containing the appropriate antibiotics to selectively count each microorganism (Table 1). Nalidixic acid combined with colistin was used to inhibit the growth of *P. aeruginosa*. Viable counts were performed after 24 h (48 h for *M. luteus*).

### 16S rRNA Fluorescence *in situ* Hybridization (FISH)

Bacteria were identified using the protocol described by Pihl et al. (2010a) with some modifications. A pan-bacteria probe [5′-HyLite 488-GCTGCCTCCCGTAGGAGT-3′ (Malic et al., 2009)] was used to detect the four microorganisms included in the model. *P. aeruginosa* and *S. aureus* were further identified by the specific probes [5′ Hylite 555-GGTAACCGTCCCCCTTGC-3′ (Pihl et al., 2010b) and 5′ ATTO 647-GAAGCAAGCTTCTCGTCCG-3′ (Lawson et al., 2011), respectively]. After carefully removing the supernatant from the Ibitreat chamber, biofilms were fixed with 4% v/v paraformaldehyde in PBS (pH 7.4) overnight at 4°C before being washed with cold sterile PBS. Bacterial biofilm cells were permeabilized using lysozyme (7 mg/mL) in 100 mM of Tris–HCl, pH 7.5, and 5 mM EDTA for 15 min at 37°C followed by lysostaphin (0.1 mg/mL) in 10 mM Tris–HCl, pH 7.5, for 5 min at 37°C. Biofilms were washed with ultrapure water and dehydrated with 50, 80, and 99% ethanol for 3 min, respectively. Wells were inoculated with 250 μL of freshly prepared hybridization buffer [0.9 M NaCl, 20 mM Tris–HCl buffer, pH 7.5, with 0.01% w/v sodium dodecyl sulphate (SDS) and 25% v/v formamide containing 50 ng/mL of the oligonucleotide probes (Eurogentec)] and incubated at 47°C for 90 min in a humid chamber. After hybridization, the Ibitreat chambers were incubated with washing buffer (20 mM Tris–HCl buffer, pH 7.5, 0.01% w/v SDS and 149 mM NaCl) for 15 min at 47°C, and then rinsed with ultrapure water.

### Measurement of Monolayer Integrity

Z-stack images of the HaCaT cells grown under different conditions stained with CellMask or CellTracker were captured using confocal fluorescence microscopy. Total fluorescence of the monolayer was used as an indirect way of measuring monolayer integrity. FIJI (free software) (Schindelin et al., 2012) enabled combination of all the slices of the Z-stack into a single plane (Z-project) in order to measure the total fluorescence emitted by the HaCat cells. Mean values of all the images taken per condition (8–10) were calculated and presented as the percentage of fluorescence compared with the HaCat control (100% fluorescence).

### Quorum Sensing Activation and Inhibition

AIP*-*1 was synthesized as previously described (Murray et al., 2014) and used to supplement *S. aureus* SH1000 Δ*agr* cultures by adding to the growth medium at a final concentration of 1 μM. ALG_QSI_ nanoparticles (Singh et al., 2019) containing 4 μg/mL of 3-amino-7-chloro-2-n-nonyl-4(3*H*)-quinazolinone (3-NH_2_-7Cl-C9-QZN) (Ilangovan et al., 2013) were added to a final concentration of 300 μg/mL at day 2 of infection, and inoculated with *P. aeruginosa*.

## Results

### Development and Validation of a Polymicrobial Biofilm Keratinocyte Colonization Model

**FIGURE 1:**
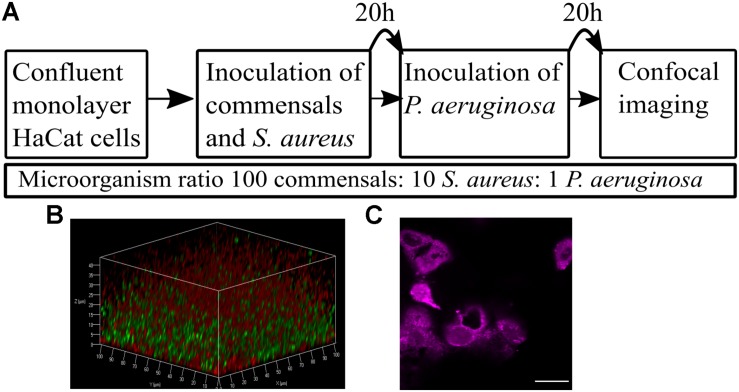
Overview of the keratinocyte polymicrobial colonization model. **(A)** The steps required to establish polymicrobial colonization of keratinocytes. **(B)** 3D projection of a dual species biofilm (red: *P. aeruginosa*-mCherry, green: *S. aureus*-GFP) set up as described in panel **A**. **(C)** Visualization of the HaCat monolayer beneath the dual species polymicrobial biofilm shown in panel **B**. Keratinocytes were stained with CellTracker and imaged with 63 × magnification. Scale bar indicates 20 μm.

To achieve co-culture of keratinocytes with more than one bacterial species, it was necessary to optimize the (i) bacterial inoculum size, (ii) timing of inoculation, and (iii) duration of co-incubation. The inoculum added to the keratinocytes (multiplicity of infection, MOI) was kept low with the aim of maintaining a healthy cell monolayer beneath the polymicrobial biofilm. To verify monolayer integrity, HaCat cells were monitored for 40 h post-inoculation with bacteria. Figure 1 illustrates the stages involved in establishing a polymicrobial species biofilm on the keratinocyte monolayer.

**FIGURE 2:**
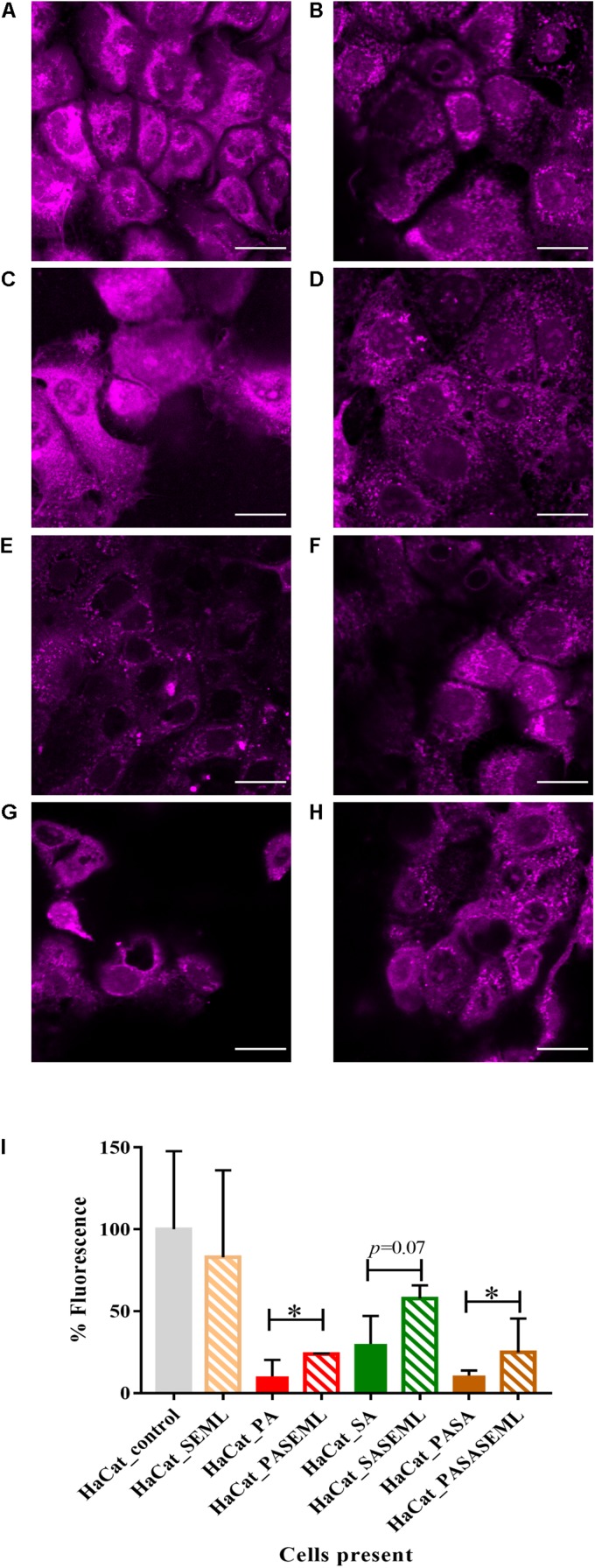
Commensals aid maintenance of monolayer integrity during pathogen colonization. HaCat monolayers were stained with CellTracker deep red prior to incubation with commensals and/or *S. aureus* for 40 h. Where present, *P. aeruginosa* was included for the final 20 h of the incubation. Bacterial inoculation ratios were as described in Figure 1, and images taken at 63 × magnification. Scale bar indicates 20 μm. HaCat cells were inoculated with **(A)** no bacteria; **(B)** commensals *S. epidermidis* and *M. luteus;* **(C)** *P. aeruginosa;* **(D)** *P. aeruginosa* and commensals; **(E)** *S. aureus;* **(F)** *S. aureus* and commensals; **(G)** *P. aeruginosa* and *S. aureus;* **(H)** HaCat *P. aeruginosa* and *S. aureus* and commensals. HaCat cells are shown as a representative single plane. **(I)** Quantification of HaCat monolayer integrity by measuring the total fluorescence of the z-stack HaCat cells using ImageJ (8–10 Z-stack images analyzed per condition, two experiments), and plotted as percentage of the HaCat only control. SE: *S. epidermidis*; ML: *M. luteus*; SA: *S. aureus*; PA: *P. aeruginosa.* ^∗^*p-*value < 0.05.

The commensals *S. epidermidis* and *M. luteus* were selected for this study due to their abundance in the healthy skin microbiota (van Rensburg et al., 2015), and similar nutritional requirements (Madigan et al., 2006). *S. aureus* and *P. aeruginosa* were chosen as representative pathogens based on their medical importance since they commonly cause skin and wound infections (Deleon et al., 2014; Serra et al., 2015). To monitor the formation of biofilms, both *S. aureus* and *P. aeruginosa* were engineered to produce a fluorescent protein by introducing a plasmid carrying the genes for either Green Fluorescent Protein (GFP) or mCherry (Table 1 and Figure 1B). The commensals were not tagged. The keratinocytes were routinely stained with CellTracker or CellMask to assess and quantify monolayer integrity (Figures 1C, 2A).

Commensal cultures were adjusted to approximately 3 × 10^3^ cfu/mL before inoculating the HaCat cell monolayer (corresponding to a final MOI = 0.005 bacteria:keratinocyte). This commensal load permitted biofilm formation without compromising HaCat cell health during the 40 h infection period (Figure 2B and Supplementary Figure S1O).

Little information is available with respect to the likely ratio of commensals to pathogens during the early stages of an infection. However, on healthy skin, the microbiota range from 10^2^–10^6^ cfu/cm^2^ (Egert and Simmering, 2016). For *P. aeruginosa*, an infecting dose for wounded skin has been reported to be ∼1,000 cfu (Leggett et al., 2012). For *S. aureus*, the infective dose was reported to be 100,000 cfu when ingestion was the route of infection (Leggett et al., 2012), but lower when the pathogen was administered topically. Thus, we assumed that the number of pathogen cells would be lower than the number of commensal cells. For the initial HaCat cell colonization, the commensal inoculum consisted of *S. epidermidis* or *M. luteus* cultures at OD_600_ 0.01 diluted 1/1000 to give a final bacterial cell number of ∼3 × 10^3^ cfu/mL in supplemented RPMI without antibiotics or phenol red. The commensals were mixed with a range of (i) *S. aureus* cells (10-fold and 50-fold dilutions of 3 × 10^3^ cfu/ml) or (ii) *P. aeruginosa* cells (10-fold, 50-fold, and 100-fold dilutions of 3 × 10^3^ cfu/ml). Both biofilm formation and HaCat health post-inoculation were monitored by confocal imaging to determine the conditions best suited for the final polymicrobial model. For *S. aureus*, the 50- and 100-fold culture dilutions did not form reproducible biofilms (data not shown). In contrast, HaCat monolayer inoculation with the 10-fold dilution of an *S. aureus* culture supported reproducible biofilm formation, although it should be noted that an ∼70% reduction in the HaCat cell monolayer integrity (calculated by measuring fluorescence as described in the Methods) was observed during the later stages (after 40 h) of colonization (Figures 2E,I and Supplementary Figure S1E). For *P. aeruginosa*, inoculation with 10- and 50-fold dilutions of culture caused complete destruction of the HaCat cells (data not shown), whereas the 100-fold dilution formed a robust biofilm while maintaining ∼10% of the HaCat monolayer integrity (Figures 2C,I and Supplementary Figure S1A).

**FIGURE 3:**
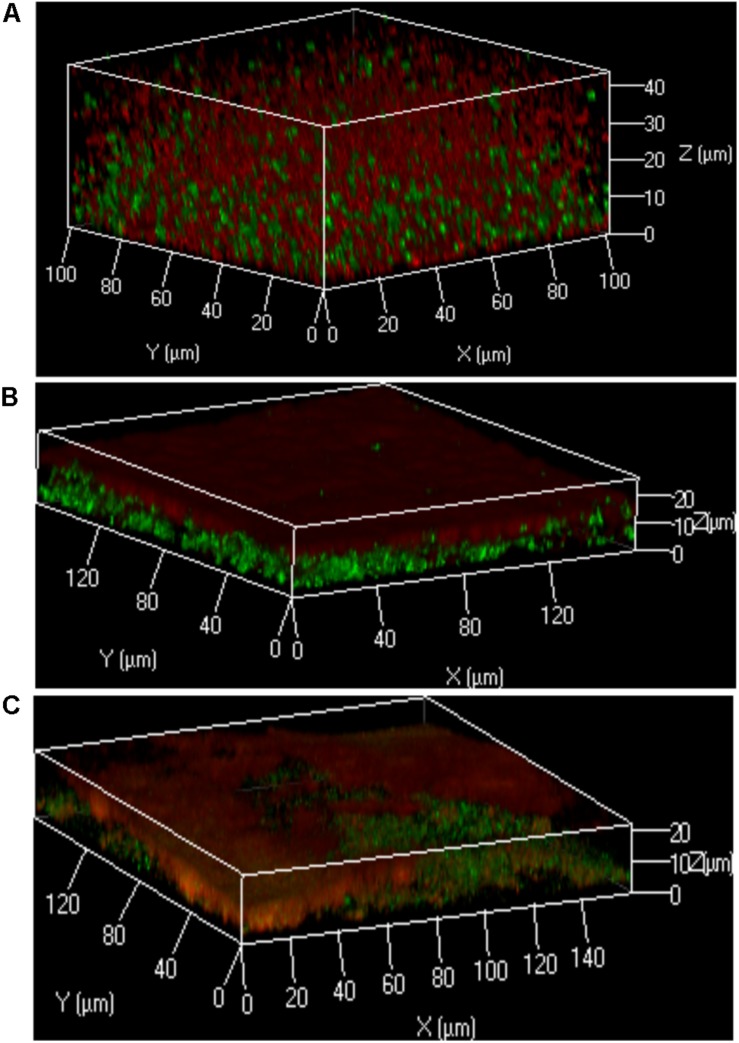
Localization of bacteria within the polymicrobial keratinocyte colonization model is species dependent. Using the protocol outlined in Figure 1, either *S. aureus, S. epidermidis* or *M. luteus* was combined with *P. aeruginosa* to form a polymicrobial biofilm on top of a HaCat monolayer. 3D reconstructions of the polymicrobial biofilms are shown using 40 × magnification. HaCat cells were not stained. **(A)** Dual species intercalated biofilm of *S. aureus* (GFP tagged, green) combined with *P. aeruginosa* (mCherry tagged, red). **(B)** Dual species layered biofilm of *S. epidermidis* (GFP tagged, green) with *P. aeruginosa* (mCherry tagged, red). **(C)** Triple species biofilm containing a layer of *M. luteus* intercalated with *S. epidermidis* between *P. aeruginosa* (above) and the keratinocyte monolayer (below). Bacteria were detected by FISH using pan-bacterial (green) and *Pseudomonas*-specific (red) probes.

**FIGURE 4:**
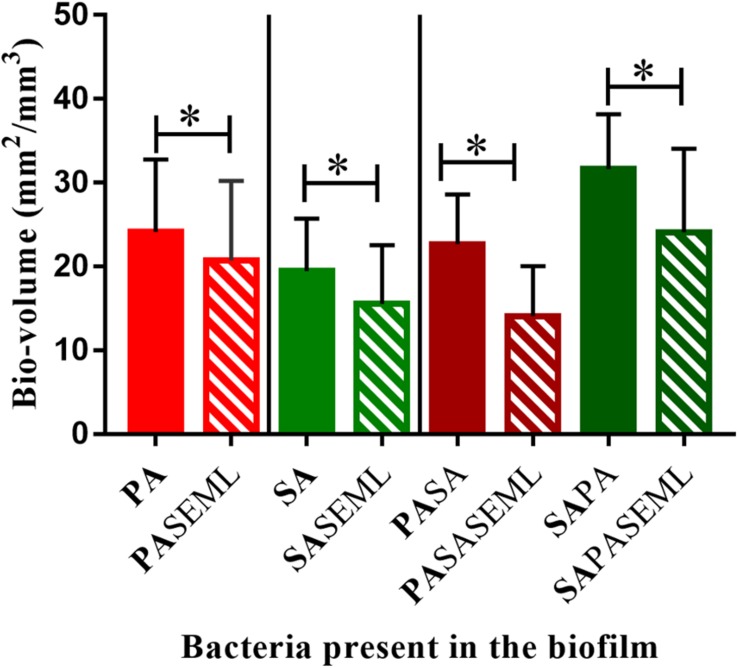
Commensals reduce biofilm biomass for both *S. aureus* and *P. aeruginosa* individually and when co-inoculated. The HaCat monolayer was inoculated with the indicated bacterial species and incubated for 40 h (*S. aureus*, commensals) or 20 h (*P. aeruginosa*) as outlined in Figure 1. The biofilm biomass was calculated using ImageJ. SE: *S. epidermidis*; ML: *M. luteus*; SA: *S. aureus*; PA: *P. aeruginosa.* Bold font indicates the bacterial species quantified. ^∗^*p-*value < 0.05. Images of the biofilms and HaCat monolayer are shown in Supplementary Figure S1, and average thickness and surface area are shown in Supplementary Figure S2.

The final bacterial ratios chosen were: 100:10:1 for commensals: *S. aureus*: *P. aeruginosa*. By pre-colonizing the HaCat cells for 20 h with commensals (and *S. aureus* if required) prior to inoculation with *P. aeruginosa* (Figure 1A), robust polymicrobial biofilms formed (Figure 3 and Supplementary Figure S1K). When quantified using COMSTAT2 for biovolume, thickness and surface area (Figure 4 and Supplementary Figure S2), the same trends were observed for each biofilm parameter quantified, confirming the stability and reproducibility of the model, although intrinsic biological variability between experiments meant that statistical significance was not always evident. However, the conditions used enabled the keratinocytes to maintain a monolayer when both pathogens were present (Figure 2H and Supplementary Figure S1K). The relative disruption of the HaCat monolayer could also be quantified following staining with CellTracker deep red (Figure 2I).

### Commensals Protect HaCat Cells From Pathogen Damage

To determine whether the commensals protected the HaCat cells from pathogen-mediated damage, the eukaryotic cells were stained using CellTracker deep red to visualize and quantify monolayer disruption (Figure 2). The penetration of CellTracker into all cellular compartments was observed in the HaCat control (Figure 2A). When the commensals were present (Figure 2B), similar cell staining was observed, indicating minimal disruption of the monolayer. Conversely, *P. aeruginosa* (Figure 2C) or *S. aureus* (Figure 2E) alone, or in combination (Figure 2G) disrupted the HaCat monolayer as shown by the reduction in the fluorescence of HaCat nuclei indicative of cell death. Interestingly, when the commensals were applied in combination with one or both pathogens, less eukaryotic cell damage was apparent (Figures 2D,F,H). To quantify these observations, the total fluorescence from HaCat cells was measured and averaged for two independent experiments (eight images each). By representing the monolayer integrity as a percentage of fluorescence relative to the HaCat control (Figure 2I), the commensals confer a significant level of protection of the keratinocytes from damage by either or both of the pathogens. In agreement with this, analysis of the polymicrobial biofilm biovolume revealed that the presence of the commensals significantly reduced the biofilm biovolume, thickness and surface coverage (Figure 4 and Supplementary Figure S2).

### Bacterial Localization Within 2D Polymicrobial Biofilms Is Species Dependent

In the keratinocyte colonization model, *S. epidermidis* and *S. aureus* showed different biofilm localization patterns when co-cultured with *P. aeruginosa* (Figure 3). Both staphylococcal species were grown for 20 h to establish their colonization of the HaCat monolayer prior to inoculation with *Pseudomonas*. Incubation was subsequently continued for a further 20 h. The microcolonies of *S. aureus* intercalated with those of *P. aeruginosa* whereas *P. aeruginosa* formed a layer on top of the *S. epidermidis* biofilm (compare Figures 3A,B). In addition, the thickness of the *P. aeruginosa/S. epidermidis* biofilm was ∼50% less than the *P. aeruginosa/S. aureus* biofilm (Figures 3A,B). The other commensal chosen for this study, *M. luteus*, behaved similarly to *S. epidermidis* by combining with the latter to form a layer between *P. aeruginosa* and the keratinocytes (Figure 3C). In Figure 3C, FISH (*in situ* Fluorescent Hybridization) rather than a fluorescent protein was used to identify the bacteria. The pan-Bacterial probe (green) and *P. aeruginosa* specific probe (red) can be observed in differentiated layers (Figure 3C) where the upper orange layer corresponds to *P. aeruginosa* alone (as both green and red probes hybridized) and the bottom layer (green) indicates the location of the commensals. The absence of green fluorescent bacteria interspersed within the orange microcolonies suggests that both commensals are co-located in a separate biofilm layer.

### Interspecies Interactions Are Detectable Within the Polymicrobial Biofilm Model

**FIGURE 5:**
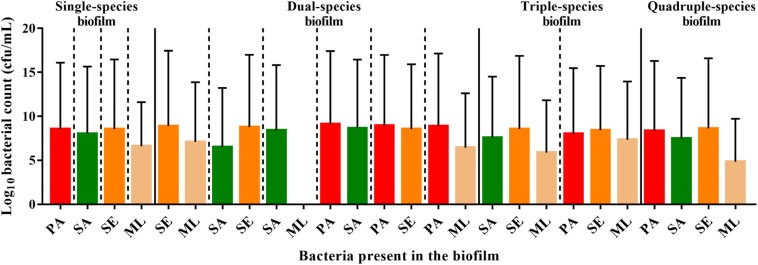
Interspecies interactions are detectable within the polymicrobial biofilm. To form a single-species biofilm, each bacterial species was grown in wells in a 96 well plate. *S. aureus* and commensals were incubated for 40 h, while *P. aeruginosa* was incubated for 20 h before harvesting to determine the number of cfu. Polymicrobial biofilms were generated by incubating *S. aureus* and commensals together for 40 h in the combinations indicated. When *P. aeruginosa* was present, it was added after 20 h, and thus was only in combination with the others for 20 h. SE: *S. epidermidis*; ML: *M. luteus*; SA: *S. aureus*; PA: *P. aeruginosa*.

The differences in the relative localization of bacteria within the polymicrobial biofilm model could derive from interactions between the bacterial species that are competitive/inhibitory or synergistic/advantageous. To assess this, viable counts for each species were quantified after growing the bacteria in single or multi-species biofilms (Figure 5).

*S. aureus*, *S. epidermidis*, and *P. aeruginosa*, exhibited comparable viable counts, irrespective of whether they were grown in monoculture or in combination (Figure 5). In contrast, the viability of *M. luteus* was completely abolished by *S. aureus* in the dual biofilm (7-fold reduction, Figure 5). Interestingly, if *S. epidermidis* was present to form a triple species biofilm, *M. luteus* cfus were not reduced, which could support either an inhibitory effect of *S. epidermidis* upon *S. aureus* or synergy between *S. epidermidis* and *M. luteus* (Figure 5). Introducing *P. aeruginosa* into the polymicrobial biofilm after 20 h to create a quadruple-species biofilm, had little effect on the viability of the Gram positive bacteria already present.

### The *S. aureus agr* QS System Contributes to HaCat Cell Damage

**FIGURE 6:**
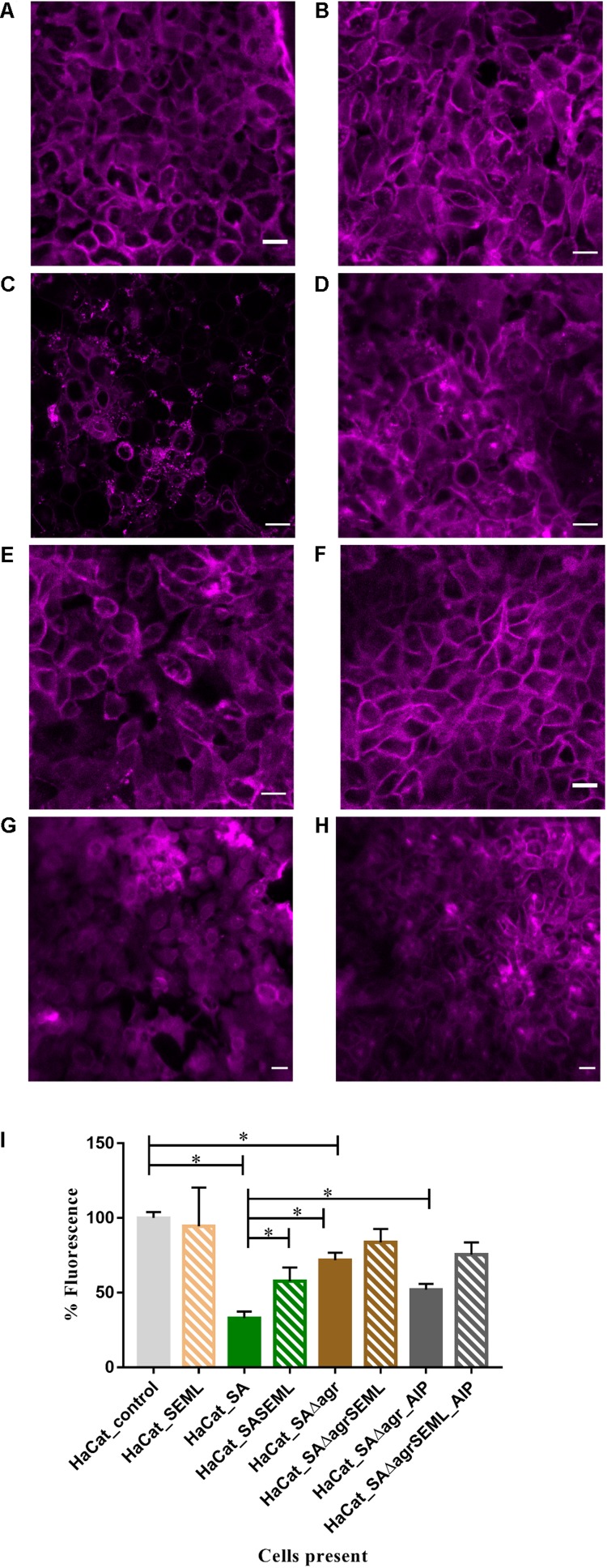
The *S. aureus agr* QS system contributes to keratinocyte cell damage HaCat cells were incubated for 40 h after inoculation with **(A)** no bacteria; **(B)** commensals *S. epidermidis* and *M. luteus*; **(C)** *S. aureus* WT; **(D)** *S. aureus* WT and commensals *S. epidermidis* and *M. luteus*; **(E)** *S. aureus*Δ*agr*; **(F)** *S. aureus*Δ*agr* and commensals *S. epidermidis* and *M. luteus*; **(G)** *S. aureus* Δ*agr* supplemented with 100 nM of AIP-1; **(H)** *S. aureus*Δ*agr* and commensals *S. epidermidis* and *M. luteus* supplemented with 100 nM of AIP-1. HaCat cells are shown as a representative single plane. **(I)** Quantification of HaCat monolayer integrity by measuring the total fluorescence of the z-stack HaCat cells using ImageJ (8–10 Z-stack images analyzed per condition, two experiments) is shown as a percentage of the control lacking bacteria. ^∗^*p-*value < 0.05. SA: *S. aureus*, SE: *S. epidermidis*; ML: *M. luteus*; AIP: autoinducing peptide. HaCat cells were stained with CellMask prior to confocal imaging. Scale bar indicates 20 μm. Images were taken at 40 × magnification.

To demonstrate the utility of the assay for exploring the mechanisms underlying HaCat monolayer damage (Figure 2E), we investigated the role of the *S. aureus agr* QS system since it regulates the expression of multiple cytotoxins including alpha-hemolysin (Murray et al., 2014). An *S. aureus* QS-deficient Δ*agr* mutant was incorporated into the keratinocyte polymicrobial model in the absence or presence of the exogenous cognate QS signaling molecule, AIP-1. Keratinocytes were stained with CellMask to determine the extent of any cell damage (Figure 6). The HaCat cell damage caused by the wild type *S. aureus* strain is clearly apparent (compare Figure 6A with Figure 6C). In contrast, the HaCat monolayer was mostly intact and comparable with the uninfected control when the cells were infected with the *S. aureus*Δ*agr* mutant (compare Figures 6C,E). When the *S. aureus*Δ*agr* mutant was supplemented with exogenous AIP-1, the keratinocyte monolayer was disrupted and rounded (dying) cells were observed, demonstrating the QS-dependent nature of the damage to the eukaryotic cells (Figure 6G). The commensals did not damage the HaCat monolayer (Figure 6B) and reduced the damage caused by both the *S. aureus* WT and the Δ*agr* mutant provided with exogenous AIP (Figures 6D,H, respectively). Quantification of the HaCat cell fluorescence confirmed these observations (Figure 6I).

### A Nanoparticle Encapsulated PQS Inhibitor Reduces *P. aeruginosa* Colonization of the Keratinocyte Polymicrobial Biofilm Model

**FIGURE 7:**
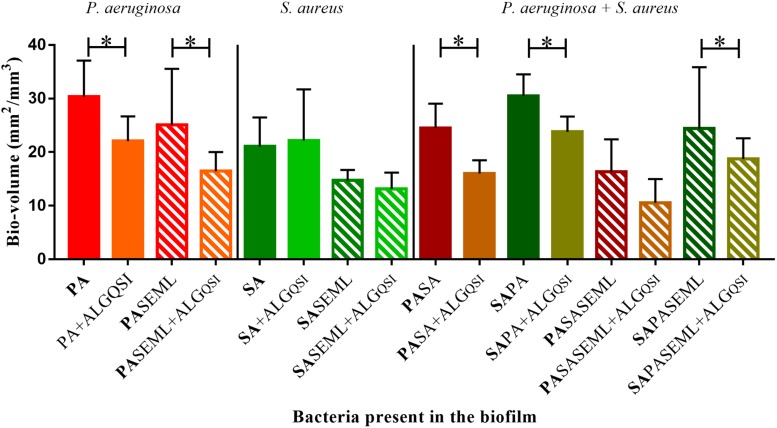
The QSI inhibitor delivered in nanoparticles (ALG_QSI_) reduced biofilm formation by *P. aeruginosa* alone and in polymicrobial biofilms. HaCat monolayers were inoculated with *S. aureus* (40 h growth) and *P. aeruginosa* (20 h growth) alone or together with commensals (as outlined in Figure 1) in the presence or absence of ALG_QSI_ nanoparticles. The biomass was quantified and plotted. SE: *S. epidermidis*; ML: *M. luteus*; SA: *S. aureus*; PA: *P. aeruginosa.* Bold font indicates the bacterial species quantified. ^∗^*p-*value < 0.05. Not all the statistically significant values have been marked. Images of the biofilms and HaCat monolayer are shown in Supplementary Figure S1, and average thickness and surface area are shown in Supplementary Figure S3.

To exemplify the use of the keratinocyte polymicrobial biofilm model for evaluating anti-virulence agents, we explored the effectiveness of alginate nanoparticles (ALG_QSI) loaded with the *P. aeruginosa* PQS system anti-virulence agent, 3-NH_2_-7Cl-C9-QZN. This was selected since we recently demonstrated that it was effective against monospecies *P. aeruginosa* biofilms in an *ex vivo* pig skin infection model (Singh et al., 2019). Figure 7 and Supplementary Figures S1, S3 show that the nanoparticles alone had no effect on *S. aureus* in monospecies biofilms or when co-colonizing with commensals. In contrast, the ALG_QSI nanoparticles reduced the *P. aeruginosa* biofilm in the presence and absence of commensals, and this was statistically significant (biovolume (*p*-value = 0.003), surface area (*p*-value = 0.0021); Figure 7 and Supplementary Figure S3). Polymicrobial biofilms containing *P. aeruginosa* and *S. aureus* in the presence of commensals were further reduced by ALG_QSI, suggesting an additive effect of nanoparticles and commensals (Figure 7 and compare Supplementary Figures S1J,L). Furthermore, the ALG_QSI nanoparticles affected the localization of *P. aeruginosa* with respect to *S aureus* (Supplementary Figure S1 compare panels **I,J** and panels **K,L**).

## Discussion

Here, we describe the development, optimization and validation of a polymicrobial biofilm keratinocyte model combining commensals and pathogens. The methodology provides a protocol for investigating the impact of biofilms upon HaCat cell monolayers using confocal fluorescence microscopy to quantify monolayer integrity. The commensals were shown to reduce pathogen-mediated damage and help protect the integrity of the underlying keratinocyte monolayer. In addition, the model was used to exemplify (a) the impact of *S. aureus* AIP-mediated QS sensing, and (b) the effectiveness of a nanoparticle QSI delivery system targeting *P. aeruginosa*. The results obtained indicate that the model offers significant potential as a screening platform to assess interventions that reduce pathogen biofilms, promote healthy skin or aid healing as well as for investigating interactions that occur between commensals, pathogens and host keratinocytes (Egert and Simmering, 2016).

In co-cultures where *P. aeruginosa* has been reported to outcompete or largely inhibit staphylococcal growth (*S. aureus* or *S. epidermidis*), comparable numbers of both bacterial species co-inoculated at the same time have generally been used (Hotterbeekx et al., 2017; Bahamondez-Canas et al., 2019). However, this approach may be unrealistic, as infective doses vary (Leggett et al., 2012), and a relatively higher number of commensals are already present in the niche (Egert and Simmering, 2016). By assessing different ratios of commensals and pathogens and by adding *P. aeruginosa* 20 h later than either *S. aureus* or *S. epidermidis*, we were able to overcome *P. aeruginosa-*mediated inhibition. Both *P. aeruginosa* and *S. aureus* are capable of outcompeting *M. luteus* in co-culture (Malic et al., 2011). Consistent with this, *M. luteus* was not recovered when co-cultured with *S. aureus* but could be recovered if allowed to establish before inoculating *P. aeruginosa*. However, when *S. epidermidis* was included, *M. luteus* survived *S. aureus* mediated-inhibition, probably due to the inhibitory effect that *S. epidermidis* has upon the *agr* QS sytem of *S. aureus* (Otto et al., 2001; Chiu et al., 2017).

The ratio of *P. aeruginosa* to *S. aureus* in the inoculum did not inhibit the formation of, or localization within dual species biofilms on HaCAT monolayers (data not shown). *S. aureus* formed microcolonies interspersed throughout the *P. aeruginosa* biofilm, as previously observed (Yang et al., 2011). However, the inoculum ratios influenced the geography of biofilms formed by *S. epidermidis* and *P. aeruginosa*. While it has been previously reported that *P. aeruginosa* outcompetes and reduces *S. epidermidis* biofilms, either when both bacteria were inoculated at the same time or when *P. aeruginosa* was added to an established *S. epidermidis* biofilm (Pihl et al., 2010b), inoculating *P. aeruginosa* at a lower cell density resulted in the formation of a biofilm consisting of two distinct layers that was ∼50% thinner than the *P. aeruginosa/S. aureus* mixed biofilm (compare Figures 3A,B).

In common with all simple experimental models and the nature of the data that can be collected from them, that described here has limitations. For example, the colony counts to enumerate viable bacteria in the biofilms may be underestimates due to microbial aggregation. To minimize this, a sonication step was incorporated (Azeredo et al., 2017). Ideally, the commensals would be labeled with fluorescent proteins with complementary emissions to avoid the washing steps required by FISH since extensive washing can disrupt biofilms. Further development of the model will also require alternative methods for assessing keratinocyte monolayer disruption and HaCat cell viability since the fluorescent stains employed offer only indirect quantification. Most of the assays available for evaluating eukaryotic cell viability can also be used for assessing bacterial viability (e.g. Alamar blue) and so are not compatible with the model. The lactate dehydrogenase assay (Koeva et al., 2017) was evaluated during the development of the current model, however, inconsistent results were obtained (data not shown) that were attributed to the complexity of the system. Direct assessment of keratinocyte viability may be possible by analysis of mRNA (Lemaître et al., 2004), however, this would be lengthy and expensive, and thus not be suitable for high-throughput screening. Since only laboratory strains were evaluated, the behavior of fresh clinical strains should be investigated.

A major limitation of the model presented that is inherent to any *in vitro* assay, is that it lacks an active host immune system or blood supply, thus it does not fully represent the *in vivo* situation (Ganesh et al., 2015; Roberts et al., 2015). Although *in vivo* models are more realistic in terms of host-biofilm interactions, they have ethical and feasibility considerations. *In vitro* models are invaluable for the preliminary testing of novel antimicrobial compounds and an irreplaceable first step for the study of the mechanisms of intercellular interactions. However, it would be desirable to corroborate the interactions described here in an *in vivo* infection model (Roberts et al., 2015).

The intricate interplay between *S. aureus* and *P. aeruginosa* (Hotterbeekx et al., 2017) likely drives the reduction of biofilm observed most markedly in the presence of commensals. However, investigation of the underlying molecular mechanisms involved remains to be undertaken. In support of our observations, *S. epidermidis* can inhibit *S. aureus* survival (Chiu et al., 2017) and *agr*-dependent QS (Otto et al., 2001). In line with this hypothesis, we report reduced keratinocyte damage using the *S. aureus*Δ*agr* mutant. This observation is supported by the previous study that described a timing dependent attenuation of cytotoxicity in ArgC mutants that produce reduced levels of AIP and suggested a survival advantage during infection by promoting colonization while restricting unnecessary overproduction of exotoxins (Sloan et al., 2019). Current studies are underway to identify the bacterial factors that contribute to the development of the polymicrobial biofilms described in the keratinocyte model presented here.

The polymicrobial keratinocyte colonization model assay was also used to assess the effectiveness of a novel *P. aeruginosa* QSI (Ilangovan et al., 2013) delivered via alginate nanoparticle encapsulation (ALG_QSI) (Singh et al., 2019). As expected, ALG_QSI reduced *P. aeruginosa* biofilm formation in monoculture. The less marked reduction compared to our previous study (Singh et al., 2019), likely reflects the shorter incubation of the bacteria with the keratinocytes. Given that the maximum reduction of pathogen biofilm occurred when the ALG_QSI was combined with the commensals, further work is required to determine whether this delivery system has any potential for treating wound infections.

In summary, we have developed a stable and reproducible keratinocyte colonization model that combines commensals and pathogens. The model can be easily adapted to study the effect of single bacterial species on HaCat cells (Singh et al., 2019) or modified by adding other commensals such as *Corynebacterium* spp., that are abundant in the skin microbiota. Alternatively it could be adapted for certain skin environments where different microbiome composition driven by skin characteristics occur (oily, moist, dry) by incorporating the most relevant bacteria or fungi (Grice and Segre, 2011). Furthermore, we have demonstrated that the ratio between bacterial species and the timing of inoculation of a polymicrobial biofilm can affect the outcome. Thus, closely mimicking the bacterial cell numbers found in a real scenario would be recommended. In the future, it will be interesting to conduct further experiments to unravel the underlying protective mechanisms that commensals provide to HaCat cells during early stage colonization by pathogens. The results obtained indicate that the model offers significant potential as a screening platform to assess interventions that reduce pathogen biofilms, promote healthy skin or aid healing as well as for investigating interactions that occur between commensals, pathogens and host keratinocytes (Egert and Simmering, 2016).

## Data Availability Statement

All datasets generated for this study are included in the article/Supplementary Material.

## Author Contributions

EJ-L, VG, AK, KH, and PW designed the experiments. EJ-L, VG, and AK performed the experiments and analyzed the data. NS developed the ALG_QSI_ nanoparticles. EJ-L, KH, and PW wrote the manuscript. EJ-L, VG, AK, NS, CA, KH, and PW critically reviewed the manuscript.

## Conflict of Interest

The authors declare that the research was conducted in the absence of any commercial or financial relationships that could be construed as a potential conflict of interest.

## References

[B1] Atkinson S. Williams P. (2009). Quorum sensing and social networking in the microbial world. *J. R. Soc. Interface* 6 959–978. 10.1098/rsif.2009.0203 19674996 PMC2827448

[B2] Azeredo J. Azevedo N. F. Briandet R. Cerca N. Coenye T. Costa A. R. (2017). Critical review on biofilm methods. *Crit. Rev. Microbiol.* 43 313–351. 10.1080/1040841X.2016.1208146 27868469

[B3] Bahamondez-Canas T. F. Heersema L. A. Smyth H. D. C. (2019). Current status of *in vitro* models and assays for susceptibility testing for wound biofilm infections. *Biomedicines* 7:34. 10.3390/biomedicines7020034 31052271 PMC6630351

[B4] Boldock E. Surewaard B. G. J. Shamarina D. Na M. Fei Y. Ali A. (2018). Human skin commensals augment *Staphylococcus aureus* pathogenesis. *Nat. Microbiol.* 3 881–890. 10.1038/s41564-018-0198-3 30013237 PMC6207346

[B5] Bosko C. A. (2019). Skin barrier insights: from bricks and mortar to molecules and microbes. *J. Drugs Dermatol.* 18 s63–s67. 30681811

[B6] Bowler P. G. Duerden B. I. Armstrong D. G. (2001). Wound microbiology and associated approaches to wound management. *Clin. Microbiol. Rev.* 14 244–269. 10.1128/CMR.14.2.244-269.2001 11292638 PMC88973

[B7] Brandwein M. Steinberg D. Meshner S. (2016). Microbial biofilms and the human skin microbiome. *NPJ Biofilms Microbiomes* 2:3. 10.1038/s41522-016-0004-z 28649397 PMC5460139

[B8] Bronesky D. Wu Z. Marzi S. Walter P. Geissmann T. Moreau K. (2016). *Staphylococcus aureus* RNAIII and its regulon link quorum sensing, stress responses, metabolic adaptation, and regulation of virulence gene expression. *Annu. Rev. Microbiol.* 70 299–316. 10.1146/annurev-micro-102215-095708 27482744

[B9] Byrd A. L. Belkaid Y. Segre J. A. (2018). The human skin microbiome. *Nat. Rev. Microbiol.* 16 143–155. 10.1038/nrmicro.2017.157 29332945

[B10] Chen Y. E. Tsao H. (2013). The skin microbiome: current perspectives and future challenges. *J. Am. Acad. Dermatol.* 69 143–155. 10.1016/j.jaad.2013.01.016 23489584 PMC3686918

[B11] Chiu L. Bazin T. Truchetet M.-E. Schaeverbeke T. Delhaes L. Pradeu T. (2017). Protective microbiota: from localized to long-reaching co-immunity. *Front. Immunol.* 8:1678. 10.3389/fimmu.2017.01678 29270167 PMC5725472

[B12] Coenye T. Nelis H. J. (2010). *In vitro* and *in vivo* model systems to study microbial biofilm formation. *J. Microbiol. Methods* 83 89–105. 10.1016/j.mimet.2010.08.018 20816706

[B13] Cogen A. L. Nizet V. Gallo R. L. (2008). Skin microbiota: a source of disease or defence? *Br. J. Dermatol.* 158 442–455. 10.1111/j.1365-2133.2008.08437.x 18275522 PMC2746716

[B14] Deleon S. Clinton A. Fowler H. Everett J. Horswill A. R. Rumbaugh K. P. (2014). Synergistic interactions of *Pseudomonas aeruginosa* and *Staphylococcus aureus* in an *in vitro* wound model. *Infect. Immun.* 82 4718–4728. 25156721 10.1128/IAI.02198-14 PMC4249327

[B15] Doherty N. Holden M. T. G. Qazi S. N. Williams P. Winzer K. (2006). Functional analysis of *luxS* in *Staphylococcus aureus* reveals a role in metabolism but not quorum sensing. *J. Bacteriol.* 188 2885–2897. 10.1128/JB.188.8.2885-2897.2006 16585750 PMC1446992

[B16] Egert M. Simmering R. (2016). The microbiota of the human skin. *Adv. Exp. Med. Biol.* 902 61–81. 10.1007/978-3-319-31248-4_5 27161351

[B17] Erin Chen Y. Fischbach M. A. Belkaid Y. (2018). Skin microbiota-host interactions. *Nature* 553 427–436. 10.1038/nature25177 29364286 PMC6075667

[B18] Galac M. R. Stam J. Maybank R. Hinkle M. Mack D. Rohde H. (2017). Complete genome sequence of *Staphylococcus epidermidis* 1457. *Genome Announc.* 5:e00450-17. 10.1128/genomeA.00450-17 PMC5454206 28572323

[B19] Ganesh K. Sinha M. Mathew-Steiner S. S. Das A. Roy S. Sen C. K. (2015). Chronic wound biofilm model. *Adv. Wound Care* 4 382–388. 10.1089/wound.2014.0587 26155380 PMC4486719

[B20] Gordon C. P. Williams P. Chan W. C. (2013). Attenuating *Staphylococcus aureus* virulence gene regulation: a medicinal chemistry perspective. *J. Med. Chem.* 56 1389–1404. 10.1021/jm3014635 23294220 PMC3585718

[B21] Grice E. A. Segre J. A. (2011). The skin microbiome. *Nat. Rev. Microbiol.* 9 244–253. 10.1038/nrmicro2537 21407241 PMC3535073

[B22] Heeb S. Itoh Y. Nishijyo T. Schnider U. Keel C. Wade J. (2000). Small, stable shuttle vectors based on the minimal pVS1 replicon for use in gram-negative, plant-associated bacteria. *Mol. Plant Microbe Interact.* 13 232–237. 10.1094/MPMI.2000.13.2.232 10659714

[B23] Heydorn A. Nielsen A. T. Hentzer M. Sternberg C. Givskov M. Ersboll B. K. (2000). Quantification of biofilm structures by the novel computer program COMSTAT. *Microbiology* 146 2395–2407. 10.1099/00221287-146-10-2395 11021916

[B24] Hotterbeekx A. Kumar-singh S. Goossens H. Maddocks S. (2017). In vivo and in vitro interactions between *Pseudomonas aeruginosa* and *Staphylococcus* spp. *Front*. *Cell. Infect*. *Microbiol*. 7:106 10.3389/fcimb.2017.00106 PMC5376567 28421166

[B25] Ilangovan A. Fletcher M. Rampioni G. Pustelny C. Rumbaugh K. Heeb S. (2013). Structural basis for native agonist and synthetic inhibitor recognition by the *Pseudomonas aeruginosa* quorum sensing regulator *PqsR* (*MvfR*). *PLoS Pathog.* 9:e1003508. 10.1371/journal.ppat.1003508 23935486 PMC3723537

[B26] Koeva M. Gutu A. D. Hebert W. Wager J. D. Yonker L. M. O’Toole G. A. (2017). An antipersister strategy for treatment of chronic *Pseudomonas aeruginosa* infections. *Antimicrob. Agents Chemother.* 61:e00987-17. 10.1128/AAC.00987-17 PMC5700368 28923873

[B27] Lawson T. S. Connally R. E. Iredell J. R. Vemulpad S. Piper J. A. (2011). Detection of *Staphylococcus aureus* with a fluorescence *in situ* hybridization that does not require lysostaphin. *J. Clin. Lab. Anal.* 25 142–147. 10.1002/jcla.20448 21438009 PMC6647721

[B28] Lee J. Wu J. Deng Y. Wang J. Wang C. Wang J. (2013). A cell-cell communication signal integrates quorum sensing and stress response. *Nat. Chem. Biol.* 9 339–343. 10.1038/nchembio.1225 23542643

[B29] Leech J. M. Dhariwala M. O. Lowe M. M. Chu K. Merana G. R. Cornuot C. (2019). Toxin-triggered interleukin-1 receptor signaling enables early-life discrimination of pathogenic versus commensal skin bacteria. *Cell Host Microbe* 26 795–809.e5. 10.1016/j.chom.2019.10.007 31784259 PMC6989301

[B30] Leggett H. C. Cornwallis C. K. West S. A. (2012). Mechanisms of pathogenesis, infective dose and virulence in human parasites. *PLoS Pathog.* 8:e1002512. 10.1371/journal.ppat.1002512 22359500 PMC3280976

[B31] Lemaître G. Lamartine J. Pitaval A. Vaigot P. Garin J. Bouet S. (2004). Expression profiling of genes and proteins in HaCaT keratinocytes: proliferating versus differentiated state. *J. Cell. Biochem.* 93 1048–1062. 10.1002/jcb.20212 15389883

[B32] Macià M. D. Rojo-Molinero E. Oliver A. (2014). Antimicrobial susceptibility testing in biofilm-growing bacteria. *Clin. Microbiol. Infect.* 20 981–990. 10.1111/1469-0691.12651 24766583

[B33] Madigan M. T. Martinko J. M. Brock T. D. (2006). *Brock Biology of Microorganisms*, 11th Edn Upper Saddle River NJ: Pearson Prentice Hall.

[B34] Malic S. Hill K. E. Hayes A. Percival S. L. Thomas D. W. Williams D. W. (2009). Detection and identification of specific bacteria in wound biofilms using peptide nucleic acid fluorescent *in situ* hybridization (PNA FISH). *Microbiology* 155 2603–2611. 10.1099/mic.0.028712-0 19477903

[B35] Malic S. Hill K. E. Playle R. Thomas D. W. Williams D. W. (2011). *In vitro* interaction of chronic wound bacteria in biofilms. *J. Wound Care* 20 569–577. 10.12968/jowc.2011.20.12.569 22240883

[B36] McVicker G. Prajsnar T. K. Foster S. J. (2018). Construction and use of *Staphylococcus aureus* strains to study within-host infection dynamics. *Methods Mol. Biol.* 1736 17–27. 10.1007/978-1-4939-7638-6_2 29322455

[B37] Monk I. R. Shah I. M. Xu M. Tan M.-W. Foster T. J. (2012). Transforming the untransformable: application of direct transformation to manipulate genetically *Staphylococcus aureus* and *Staphylococcus epidermidis*. *mBio* 3:e00277-11. 10.1128/mBio.00277-11 PMC3312211 22434850

[B38] Murray E. J. Crowley R. C. Truman A. Clarke S. R. Cottam J. A. Jadhav G. P. (2014). Targeting *Staphylococcus aureus* quorum sensing with nonpeptidic small molecule inhibitors. *J. Med. Chem.* 57 2813–2819. 10.1021/jm500215s 24592914 PMC4010551

[B39] Negrini T. C. Arthur R. A. Waeiss R. A. Carlosa I. Z. Srinivasan M. (2014). Salivary epithelial cells as model to study immune response against cutaneous pathogens. *Clin. Transl. Sci.* 7 48–51. 10.1111/cts.12113 24118988 PMC5414475

[B40] Njoroge J. Sperandio V. (2009). Jamming bacterial communication: new approaches for the treatment of infectious diseases. *EMBO Mol. Med.* 1 201–210. 10.1002/emmm.200900032 20049722 PMC2801573

[B41] Novick R. P. Ross H. F. Projan S. J. Kornblum J. Kreiswirth B. Moghazeh S. (1993). Synthesis of staphylococcal virulence factors is controlled by a regulatory RNA molecule. *EMBO J.* 12 3967–3975. 10.1002/j.1460-2075.1993.tb06074.x 7691599 PMC413679

[B42] Ortori C. A. Dubern J.-F. Chhabra S. R. Cámara M. Hardie K. Williams P. (2011). Simultaneous quantitative profiling of N-acyl-L-homoserine lactone and 2-alkyl-4(1H)-quinolone families of quorum-sensing signaling molecules using LC-MS/MS. *Anal. Bioanal. Chem.* 399 839–850. 10.1007/s00216-010-4341-0 21046079

[B43] Otto M. Echner H. Voelter W. Gotz F. (2001). Pheromone cross-inhibition between *Staphylococcus aureus* and *Staphylococcus epidermidis*. *Infect. Immun.* 69 1957–1960. 10.1128/IAI.69.3.1957 11179383 PMC98112

[B44] Parlet C. P. Brown M. M. Horswill A. R. (2019). Commensal staphylococci influence *Staphylococcus aureus* skin colonization and disease. *Trends Microbiol.* 27 497–507. 10.1016/j.tim.2019.01.008 30846311 PMC7176043

[B45] Percival S. L. Hill K. E. Williams D. W. Hooper S. J. Thomas D. W. Costerton J. W. (2012). A review of the scientific evidence for biofilms in wounds. *Wound Repair. Regen.* 20 647–657. 10.1111/j.1524-475X.2012.00836.x 22985037

[B46] Peters B. M. Jabra-rizk M. A. Costerton J. W. Shirtliff M. E. (2012). Polymicrobial interactions: impact on pathogenesis and human disease. *Clin. Microbiol. Rev.* 25 193–213. 10.1128/cmr.00013-11 22232376 PMC3255964

[B47] Pihl M. Chávez de Paz L. E. Schmidtchen A. Svensäter G. Davies J. R. (2010a). Effects of clinical isolates of *Pseudomonas aeruginosa* on *Staphylococcus epidermidis* biofilm formation. *FEMS Immunol. Med. Microbiol.* 59 504–512. 10.1111/j.1574-695X.2010.00707.x 20579097

[B48] Pihl M. Davies J. R. Chávez de Paz L. E. Svensäter G. (2010b). Differential effects of *Pseudomonas aeruginosa* on biofilm formation by different strains of *Staphylococcus epidermidis*. *FEMS Immunol. Med. Microbiol.* 59 439–446. 10.1111/j.1574-695X.2010.00697.x 20528934

[B49] Qazi S. N. A. Counil E. Morrissey J. Rees C. E. D. Cockayne A. Winzer K. (2001). *agr* expression precedes escape of internalized *Staphylococcus aureus* from the host endosome. *Infect. Immun.* 69 7074–7082. 10.1128/IAI.69.11.7074-7082.2001 11598083 PMC100088

[B50] Roberts A. E. L. Kragh K. N. Bjarnsholt T. Diggle S. P. (2015). The limitations of in vitro experimentation in understanding biofilms and chronic infection. *J. Mol. Biol.* 427 3646–3661. 10.1016/j.jmb.2015.09.002 26344834

[B51] Rokem J. S. Vongsangnak W. Nielsen J. (2011). Comparative metabolic capabilities for *Micrococcus luteus* NCTC 2665, the “Fleming” strain, and actinobacteria. *Biotechnol. Bioeng.* 108 2770–2775. 10.1002/bit.23212 21618466

[B52] Romero M. Acuna L. Otero A. (2012). Patents on quorum quenching: interfering with bacterial communication as a strategy to fight infections. *Recent Pat. Biotechnol.* 6 2–12. 10.2174/187220812799789208 22420877

[B53] Schindelin J. Arganda-Carreras I. Frise E. Kaynig V. Longair M. Pietzsch T. (2012). Fiji: an open-source platform for biological-image analysis. *Nat. Methods* 9 676–682. 10.1038/nmeth.2019 22743772 PMC3855844

[B54] Serra R. Grande R. Butrico L. Rossi A. Settimio U. F. Caroleo B. (2015). Chronic wound infections: the role of *Pseudomonas aeruginosa* and *Staphylococcus aureus*. *Expert Rev. Anti Infect. Ther.* 13 605–613. 10.1586/14787210.2015.1023291 25746414

[B55] Shaaban M. Elgaml A. Habib E. S. E. (2019). Biotechnological applications of quorum sensing inhibition as novel therapeutic strategies for multidrug resistant pathogens. *Microb. Pathog.* 127 138–143. 10.1016/j.micpath.2018.11.043 30503958

[B56] Singh N. Romero M. Travanut A. Monteiro P. F. Jordana-Lluch E. Hardie K. R. (2019). Dual bioresponsive antibiotic and quorum sensing inhibitor combination nanoparticles for treatment of *Pseudomonas aeruginosa* biofilms in vitro and ex vivo. *Biomater. Sci.* 7 4099–4111. 10.1039/c9bm00773c 31355397

[B57] Sloan T. J. Murray E. Yokoyama M. Massey R. C. Chan W. C. Bonev B. B. (2019). Timing is everything: impact of naturally occurring *Staphylococcus aureus AgrC* cytoplasmic domain adaptive mutations on autoinduction. *J. Bacteriol.* 201:e00409-19. 10.1128/JB.00409-19 PMC6755735 31358609

[B58] Snyder R. J. Bohn G. Hanft J. Harkless L. Kim P. Lavery L. (2017). Wound biofilm: current perspectives and strategies on biofilm disruption and treatments. *Wounds* 29 S1–S17. 28682297

[B59] Soukarieh F. Williams P. Stocks M. J. Cámara M. (2018). *Pseudomonas aeruginosa* quorum sensing systems as drug discovery targets: current position and future perspectives. *J. Med. Chem.* 61 10385–10402. 10.1021/acs.jmedchem.8b00540 29999316

[B60] van Rensburg J. J. Lin H. Gao X. Toh E. Fortney K. R. Ellinger S. (2015). The human skin microbiome associates with the outcome of and is influenced by bacterial infection. *mBio* 6:e01315-15. 10.1128/mBio.01315-15 PMC4600114 26374122

[B61] Yang L. Liu Y. Markussen T. Høiby N. Tolker-Nielsen T. Molin S. (2011). Pattern differentiation in co-culture biofilms formed by *Staphylococcus aureus* and *Pseudomonas aeruginosa*. *FEMS Immunol. Med. Microbiol.* 62 339–347. 10.1111/j.1574-695X.2011.00820.x 21595754

