# Supplementary material for: A Simple Polymicrobial Biofilm Keratinocyte Colonization Model for Exploring Interactions Between Commensals, Pathogens and Antimicrobials
Source: Front Microbiol. 2020 Feb 26;11:291. doi: 10.3389/fmicb.2020.00291 (PMC7054238; doi:10.3389/fmicb.2020.00291)

Supplementary material

Figure S1. Representative biofilms and HaCat cell monolayers in the presence and absence of a nanopartlicle delivered QSI (ALG_{QSI}). Keratinocyte colonisation was undertaken as outlined in **Figure 1**. After colonisation (40h for commensals and *S. aureus*, 20h for *P. aeruginosa*) the ALG_QSI was applied where indicated. **(A)** HaCat + PA; **(B)** HaCat + PA+ALG_QSI; **(C)** HaCat + PASEML; **(D)** HaCat + PASEML+ALG_QSI; **(E)** HaCat + SA; **(F)** HaCat + SA+ALG_QSI; **(G)** HaCat + SASEML; **(H)** HaCat + SASEML+ALG_QSI; **(I)** HaCat + PASA; **(J)** HaCat + PASA+ALG_QSI; **(K)** HaCat + PASASEML; **(L)** HaCat + PASASEML+ALG_QSI; **(M)** HaCat; **(N)** HaCat + ALG_QSI; **(O)** HaCat + SEML; **(P)** HaCat + SEML + ALG_QSI. SE: *S. epidermidis*; ML: *M. luteus*; SA: *S. aureus*; PA: *P. aeruginosa.* 63x magnification. HaCat cells were stained prior to infection with CellTracker (magenta). Green: *S. aureus*; red: *P. aeruginosa.* Scale bar: 20 μm.

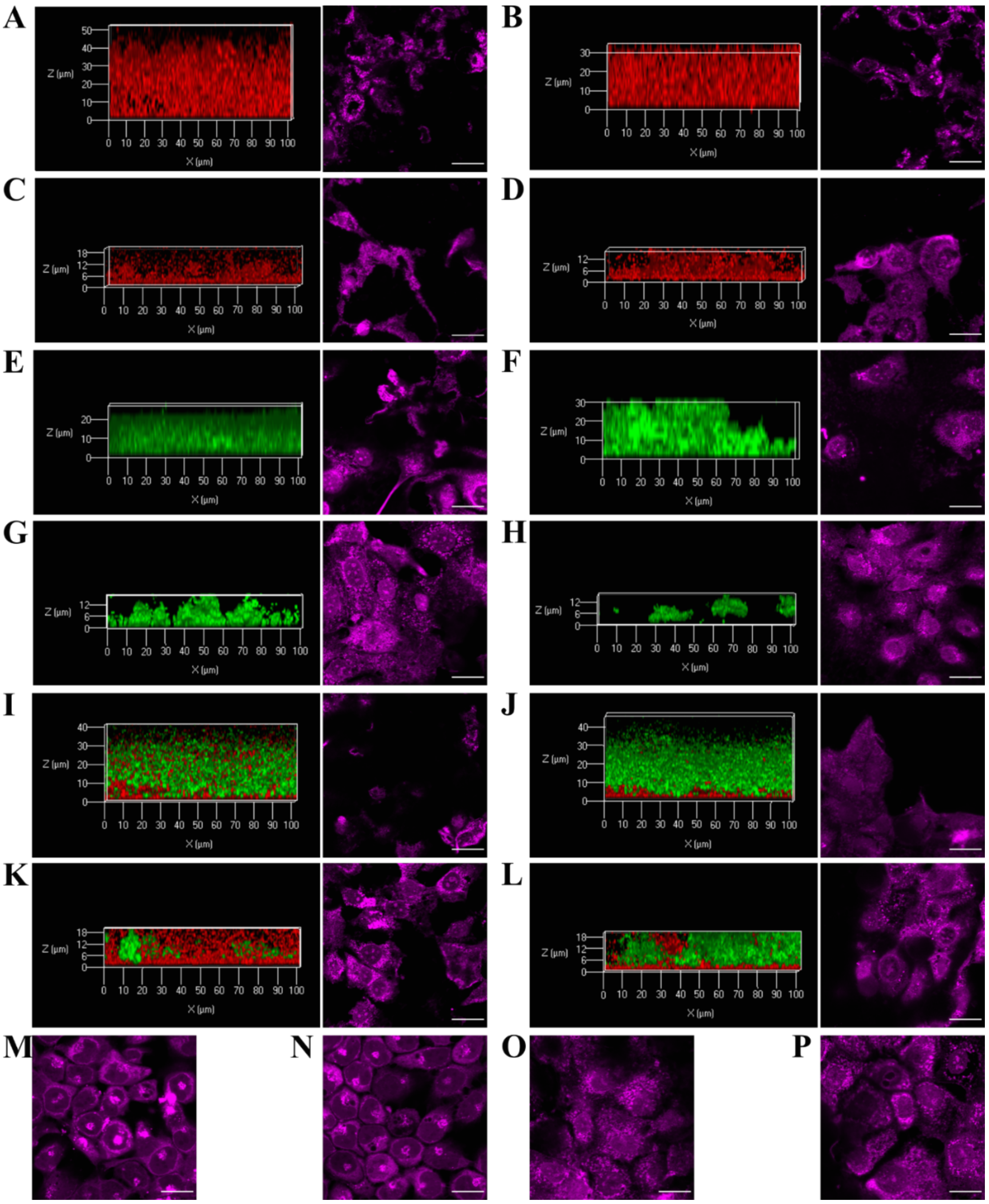

Supplementary Figure S2. Quantification of average thickness and surface area verify the reproducibility of the keratinocyte polymicrobial biofilm colonisation model. The HaCat monolayer was inoculated with the indicated bacteria and incubated for 40h (*S. aureus*, commensals) or 20h (*P. aeruginosa*). The **(A)** biofilm average thickness, and **(B)** surface area were quantified and plotted. SE: *S. epidermidis*; ML: *M. luteus*; SA: *S. aureus*; PA: *P. aeruginosa.* Bold font indicates the bacterial species quantified. * *p*-value < 0.05. Images of the biofilms and HaCat monolayer are shown in **Figure S1** and biomass quantification in **Figure 4**.

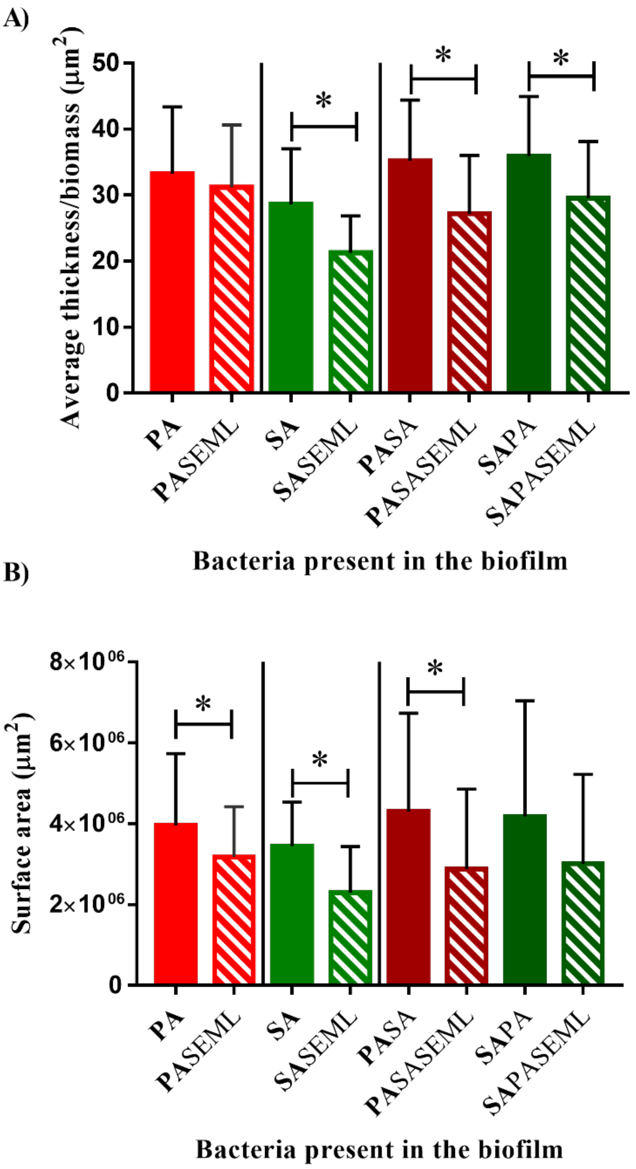

Supplementary Figure S3. The nanoparticle encapsulated QSI (ALG$_{QSI}$) reduced biofilm formation by *P. aeruginosa* alone and in polymicrobial culture HaCat monolayers were inoculated with *S. aureus* (40h incubation) and *P. aeruginosa* (20h incubation) alone or together with or without commensals (as outlined in **Figure 1**) in the presence or absence of ALG$_{QSI}$ nanoparticles. The **(A)** average thickness and **(B)** surface area were quantified and plotted. SE: *S. epidermidis*; ML: *M. luteus*; SA: *S. aureus*; PA: *P. aeruginosa.* Bold font indicates the bacterial species quantified. * *p*-value < 0.05. Not all the statistically significant values have been marked. Images of the biofilms and HaCat monolayer is shown in **Figure S1**, and Biomass quantification is shown in **Figure 7**.

A)

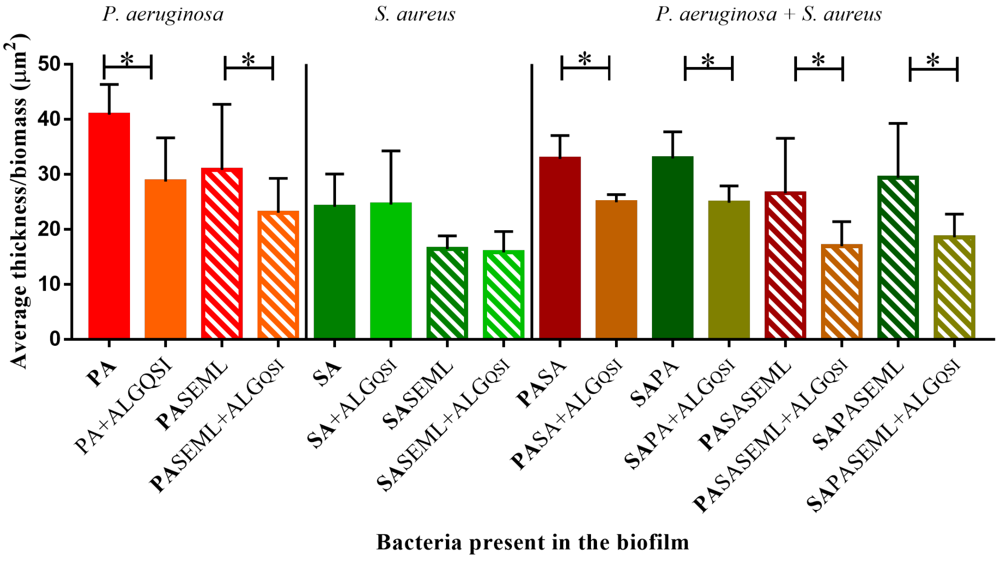

B)

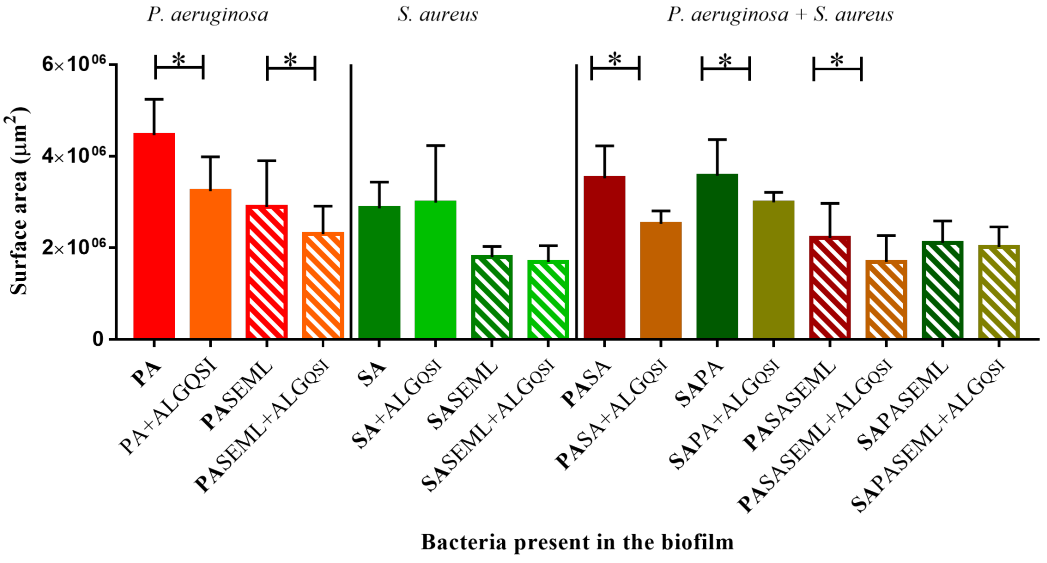

Supplement: Supplementary file 1 [file Data_Sheet_1.PDF]
